# Supplementary figures and images for: Replicating prediction algorithms for hospitalization and corticosteroid use in patients with inflammatory bowel disease
Source: PLoS One. 2021 Sep 20;16(9):e0257520. doi: 10.1371/journal.pone.0257520 (PMC8452029; doi:10.1371/journal.pone.0257520)

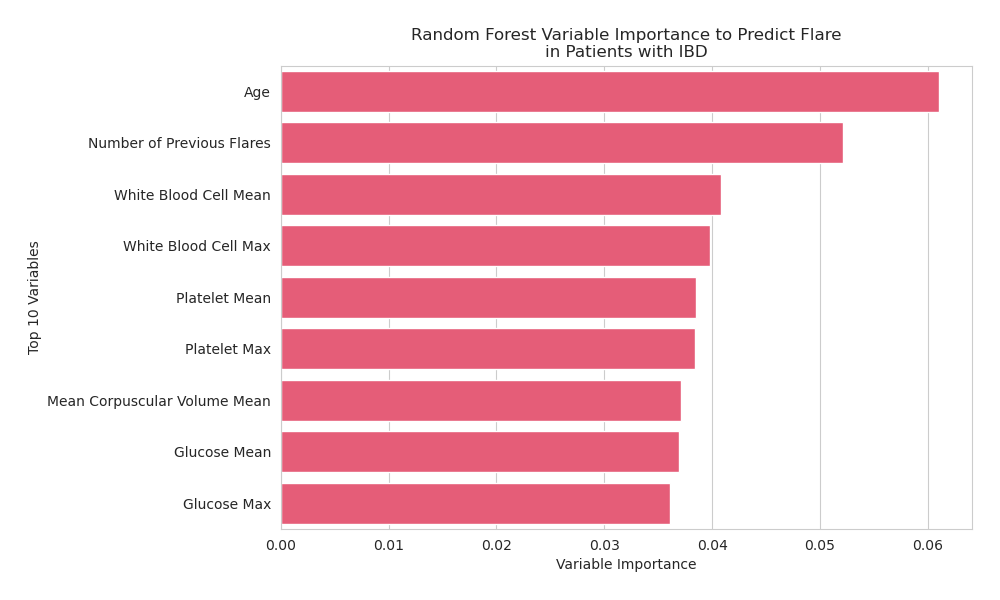

Supplement: S1 Fig — IBD = inflammatory bowel disease; Max = maximum. (TIFF) [file pone.0257520.s001.tiff]

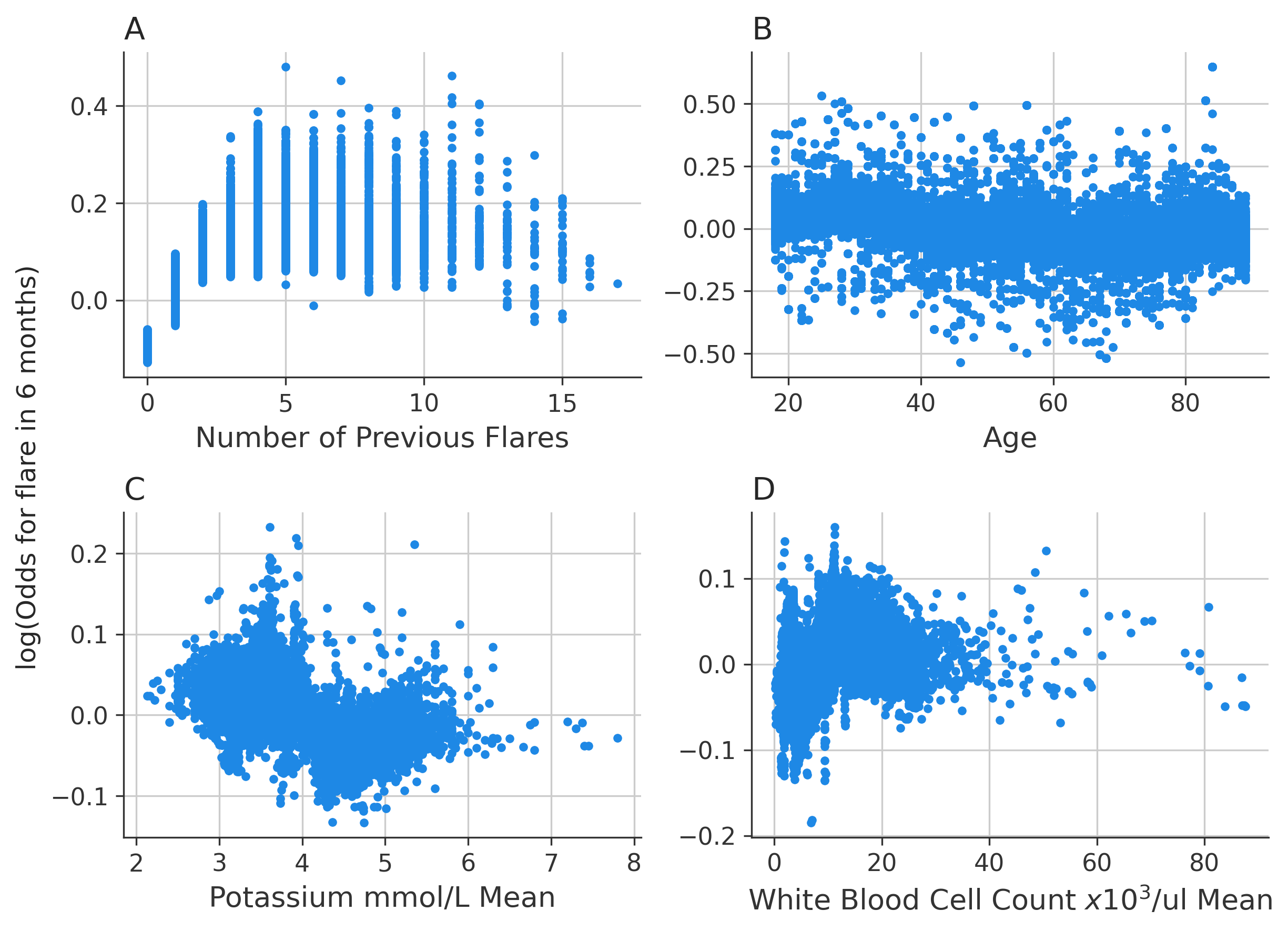

Supplement: S2 Fig — Dependency Plot For A) Number of Previous Flares, B) Age At Visit, C) Potassium Mmol/L Past Visits Mean, and D) WBC Count×103/μL Past Visits Mean. (TIFF) [file pone.0257520.s002.tiff]

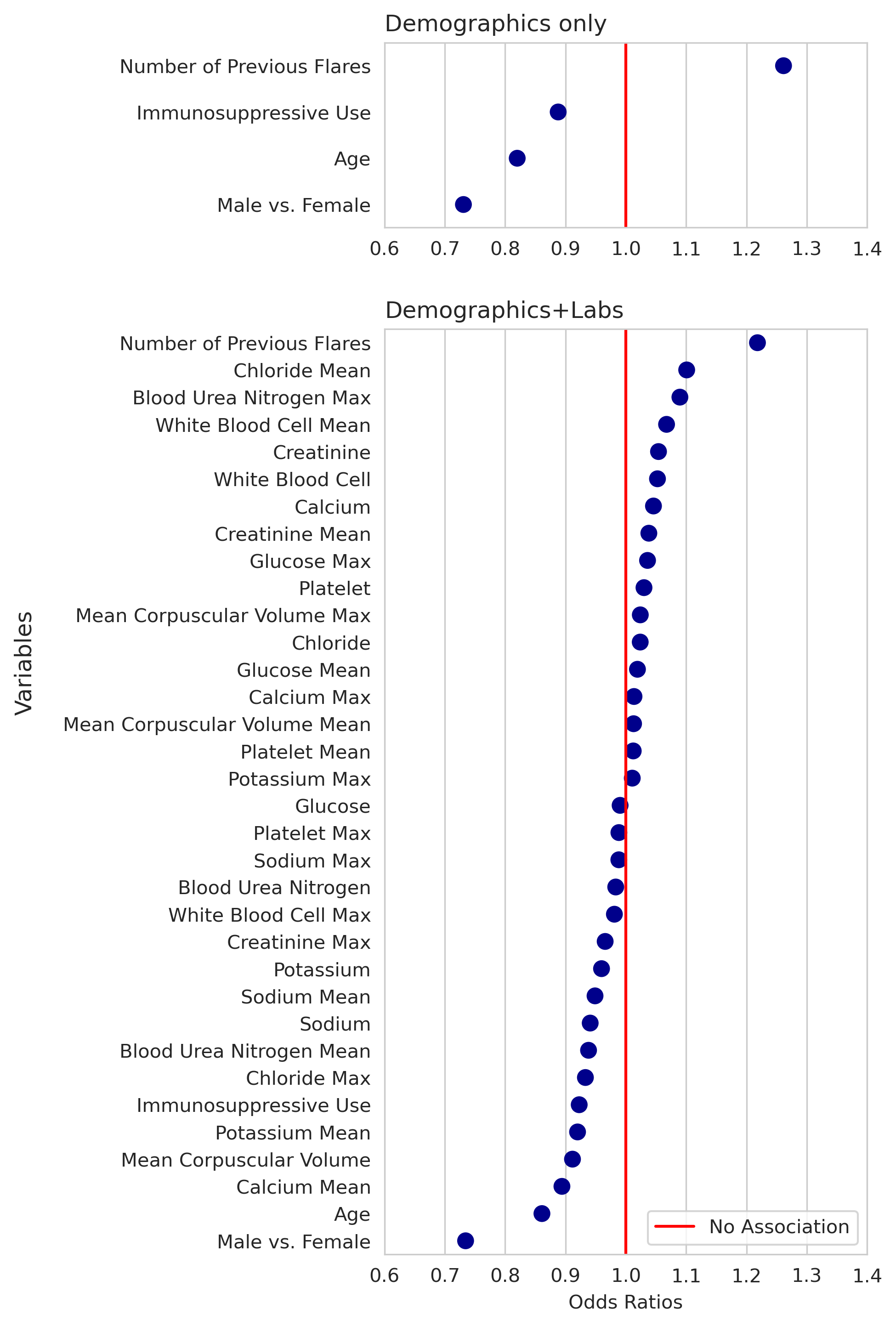

Supplement: S3 Fig — Max = maximum; Labs = laboratory variables. Note: Python scikit-learn used for logistic models does not estimate variance parameters for the coefficients and are therefore not reported. (TIFF) [file pone.0257520.s003.tiff]

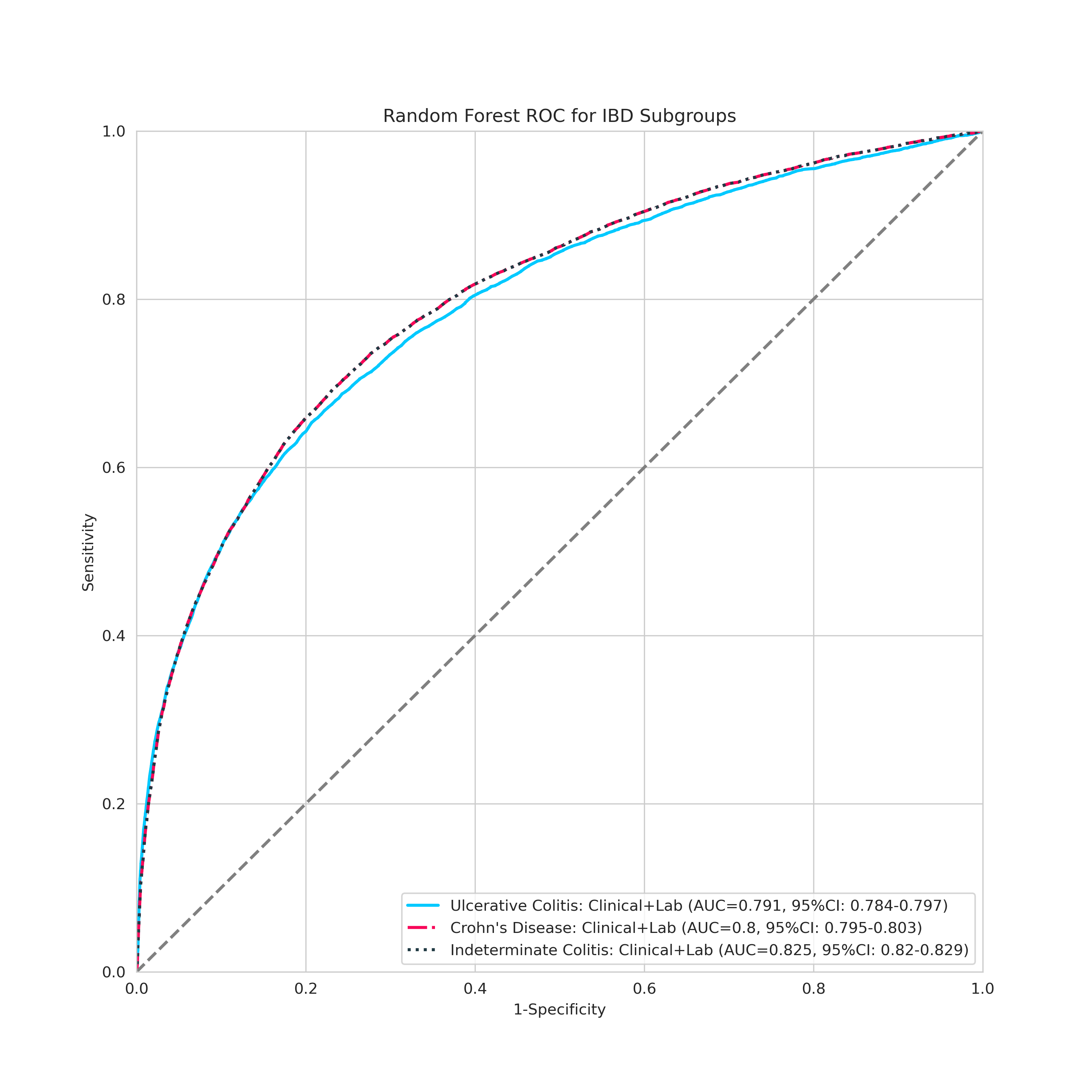

Supplement: S4 Fig — AUC = area under the curve; auROC = area under the receiver operating curve; RF = regression model; ROC = receiver operating curve. (TIFF) [file pone.0257520.s004.tiff]

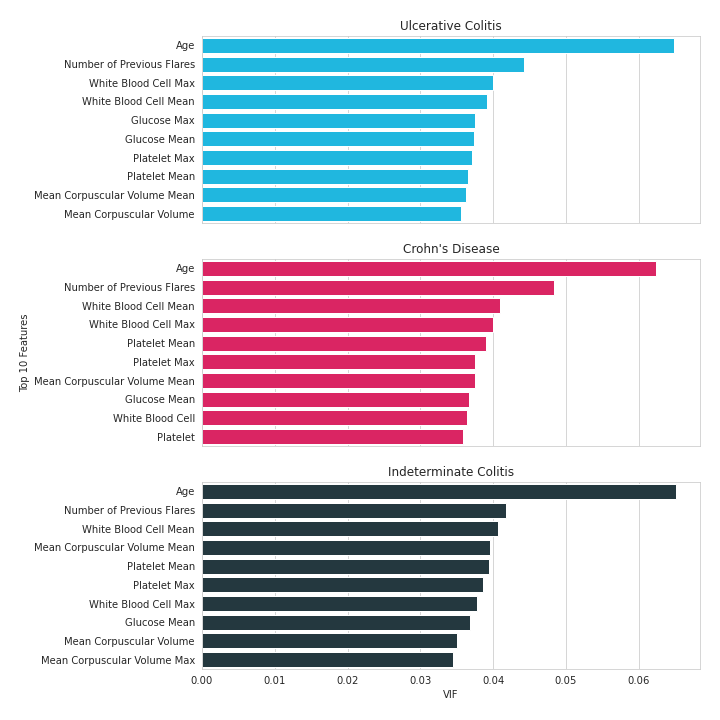

Supplement: S5 Fig — Max = maximum. (TIFF) [file pone.0257520.s005.tiff]
