# Supplementary material for: Replicating prediction algorithms for hospitalization and corticosteroid use in patients with inflammatory bowel disease
Source: PLoS One. 2021 Sep 20;16(9):e0257520. doi: 10.1371/journal.pone.0257520 (PMC8452029; doi:10.1371/journal.pone.0257520)
Supplement: S1 Table — (DOCX) [file pone.0257520.s006.docx]

S1 Table. Patient variables.

| Variable Groups | Description |
| --- | --- |
| Demographic Variables: | Age, sex, number of previous flares (number of previous outpatient corticosteroids or hospitalizations), any immunosuppressive (thiopurine, methotrexate, anti-tumor necrosis factor (TNF), or combination therapy) |
| Laboratory Variables: | White blood cell (WBC) count, hemoglobin, hematocrit (HCT), mean corpuscular volume (MCV), mean corpuscular hemoglobin concentrate (MCHC), platelets, sodium, potassium, glucose, blood urea nitrogen (BUN), serum creatinine, calcium, bicarbonate, chloride, albumin, aspartate aminotransferase (AST), alanine aminotransferase( ALT), total protein, alkaline phosphatase (ALP), bilirubin  For a given patient, the mean and maximum of past laboratory measurements prior to visit were calculated |
