# Supplementary material for: Replicating prediction algorithms for hospitalization and corticosteroid use in patients with inflammatory bowel disease
Source: PLoS One. 2021 Sep 20;16(9):e0257520. doi: 10.1371/journal.pone.0257520 (PMC8452029; doi:10.1371/journal.pone.0257520)
Supplement: S2 Table — (DOCX) [file pone.0257520.s007.docx]

**S2 Table.** Bootstrapped estimates of median and 95% confidence interval (CI) model performance for random forest (RF) model with clinical and multiple imputation by chain equation laboratory features on laboratory variables measured at >70% of visits.

| Diagnostic Metric | Median (50%) | Lower 95% | Upper 95% |
| --- | --- | --- | --- |
| Sensitivity | 0.597 | 0.591 | 0.602 |
| Specificity | 0.730 | 0.728 | 0.733 |
| Positive predictive value | 0.239 | 0.236 | 0.242 |
| Negative predictive value | 0.928 | 0.926 | 0.929 |
| Accuracy | 0.714 | 0.712 | 0.716 |
| Area under the receiver operator curve | 0.727 | 0.724 | 0.730 |

RF = regression model.
