# Supplementary material for: Replicating prediction algorithms for hospitalization and corticosteroid use in patients with inflammatory bowel disease
Source: PLoS One. 2021 Sep 20;16(9):e0257520. doi: 10.1371/journal.pone.0257520 (PMC8452029; doi:10.1371/journal.pone.0257520)
Supplement: S1 List — (DOCX) [file pone.0257520.s009.docx]

**S1 List.** Generic names of corticosteroids

Types of corticosteroids searched for included: prednisone, methylprednisolone, hydrocortisone, cortisone, tixocortol pivalate, prednisolone, budesonide, pivalone, or methylprednisone.
