# Supplementary material for: Replicating prediction algorithms for hospitalization and corticosteroid use in patients with inflammatory bowel disease
Source: PLoS One. 2021 Sep 20;16(9):e0257520. doi: 10.1371/journal.pone.0257520 (PMC8452029; doi:10.1371/journal.pone.0257520)
Supplement: S2 List — (DOCX) [file pone.0257520.s010.docx]

**S2 List.** ICD-9-CM codes for a variety of common inflammatory comorbid conditions

Excluded comorbidities based on International Classification of Diseases, 9th Revision, Clinical Modification (ICD‑9‑CM) codes within 7 days of a corticosteroid prescription (491.x, 492.x, 496.x for chronic obstructive pulmonary disease, 493.x for asthma, 714.x for rheumatoid arthritis, 710.x for lupus, 69x.x for dermatitis, 490.x for bronchitis, 461.x and 473.x for sinusitis, 472.x and 477.x for rhinitis, 725.x for polymyalgia rheumatica, 339.x for cluster headaches, 571.42 and for autoimmune hepatitis, 339.x for cluster headaches occurring within 7 days of a fill were used to identify corticosteroid fills for non‑IBD/other conditions [OS]).
