# Supplementary material for: Replicating prediction algorithms for hospitalization and corticosteroid use in patients with inflammatory bowel disease
Source: PLoS One. 2021 Sep 20;16(9):e0257520. doi: 10.1371/journal.pone.0257520 (PMC8452029; doi:10.1371/journal.pone.0257520)
Supplement: Attachment — Submitted filename: Response to Reviewers.docx [file pone.0257520.s011.docx]

**Response to edits requested on submission PONE-D-21-20616R1**:

***We've checked your submission and before we can proceed, we need you to address the following issues:

1. Thank you for stating the following in the Competing Interests section: 
[I have read the journal's policy and the authors of this manuscript have the following competing interests:
Ryan Gan and Diana Sun are full time employees of Genentech, Inc., a member of the Roche group, and own shares of Roche stock.
Amanda Tatro is a full time employee of F. Hoffmann La Roche AG and own shares of Roche stock.].    
We note that one or more of the authors are employed by a commercial company: Roche stock.*** 

***a) Please provide an amended Funding Statement declaring this commercial affiliation, as well as a statement regarding the Role of Funders in your study. If the funding organization did not play a role in the study design, data collection and analysis, decision to publish, or preparation of the manuscript and only provided financial support in the form of authors' salaries and/or research materials, please review your statements relating to the author contributions, and ensure you have specifically and accurately indicated the role(s) that these authors had in your study. You can update author roles in the Author Contributions section of the online submission form.***

Amended Funding Statement provided in updated Cover Letter. The funding organization only provided financial support in the form of authors’ salaries and/or research materials and is reflected as such in the Author Contributions.

***Please also include the following statement within your amended Funding Statement. 
“The funder provided support in the form of salaries for authors [insert relevant initials], but did not have any additional role in the study design, data collection and analysis, decision to publish, or preparation of the manuscript. The specific roles of these authors are articulated in the ‘author contributions’ section.”
If your commercial affiliation did play a role in your study, please state and explain this role within your updated Funding Statement.***

This statement has been included in our amended Funding Statement within the updated Cover Letter.

***b) Please also provide an updated Competing Interests Statement declaring this commercial affiliation along with any other relevant declarations relating to employment, consultancy, patents, products in development, or marketed products, etc.  

Within your Competing Interests Statement, please confirm that this commercial affiliation does not alter your adherence to all PLOS ONE policies on sharing data and materials by including the following statement: "This does not alter our adherence to  PLOS ONE policies on sharing data and materials.” (as detailed online in our guide for authors*** [***http://journals.plos.org/plosone/s/competing-interests***](http://journals.plos.org/plosone/s/competing-interests)***) . If this adherence statement is not accurate and  there are restrictions on sharing of data and/or materials, please state these. Please note that we cannot proceed with consideration of your article until this information has been declared.

Please include both an updated Funding Statement and Competing Interests Statement in your cover letter. We will change the online submission form on your behalf.***

We have updated our Competing Interests statement in our Cover Letter adding the following statement “This does not alter our adherence to PLOS ONE policies on data sharing and materials.”


***2. Please remove your Supporting Information figures within your manuscript file, leaving only the individual TIFF/EPS image files.  These will be automatically included in the reviewer’s PDF***

We have removed Supporting Information figures within the manuscript file, only leaving the figure captions.

***3. We note your current Data Availability statement is:

"The Optum EHR [5] data used in this study were licensed from Optum and are not publicly available due to data licensing and use agreements; interested researchers can contact Optum to license the data. All interested researchers can access the data in the same manner as the authors. The authors had no special access privileges. Optum EHR contact website:*** [***https://www.optum.com/business/solutions/government/federal/data-analytics-federal/clinical-data.html***](https://www.optum.com/business/solutions/government/federal/data-analytics-federal/clinical-data.html)***.
R code (GitHub:*** [***https://github.com/CCMRcodes/IBD_Flare***](https://github.com/CCMRcodes/IBD_Flare)***) and paper previously published by Waljee et al.[3] was reviewed and adapted to Python code.
Manuscript code and summary data used to produce tables and plots can be found in the public GitHub repository:*** [***https://github.com/phcanalytics/ibd_flare_model***](https://github.com/phcanalytics/ibd_flare_model)***. Note, SHAP values require raw data to be calculated and are not included in the repository due to data use agreements.
Transparent Reporting of a Multivariable Prediction Model for Individual Prognosis or Diagnosis (TRIPOD) guidelines were implemented;[24] checklist can be found in S3 Table."

PLOS defines the "minimal data set" as consisting of the data used to reach the conclusions drawn in the manuscript with related metadata and methods, and any additional data required to replicate the reported study findings in their entirety. This includes:
- The values behind the means, standard deviations and other measures reported;
- The values used to build graphs;
- The points extracted from images for analysis.

Before we proceed, please address the following prompts:

a.) Please confirm whether your current Data Availability statement includes all of the relevant information and links needed for accessing the minimal data set necessary for replicating the study's results?***

We confirm that our Data Availability statements includes all relevant information and links need for accessing the minimal data set necessary for replicating the study’s results. In an effort to improve data accessibility, we have put a markdown table on meta data used to make the plots and tables in the results folder on the public git repository and modified the data availability paragraph slightly to separate out the link for the summary data availability. We have updated our Data Availability statement in the manuscript slightly to read “ The Optum EHR [5] data used in this study were licensed from Optum and are not publicly available due to data licensing and use agreements; interested researchers can contact Optum to license the data. All interested researchers can access the data in the same manner as the authors. The authors had no special access privileges. Optum EHR contact website: <https://www.optum.com/business/solutions/government/federal/data-analytics-federal/clinical-data.html>.

R code (GitHub: https://github.com/CCMRcodes/IBD_Flare) and paper previously published by Waljee et al.[3] was reviewed and adapted to Python code.

Manuscript code used to produce tables and plots can be found in the public GitHub repository: <https://github.com/phcanalytics/ibd_flare_model>. Summary data used to create plots and tables can be found in the public GitHub repository: <https://github.com/phcanalytics/ibd_flare_model/tree/master/results>. Note, SHAP values require raw data to be calculated and are not included in the repository due to data use agreements.

Transparent Reporting of a Multivariable Prediction Model for Individual Prognosis or Diagnosis (TRIPOD) guidelines were implemented;[24] checklist can be found in S3 Table.”

***We will update your Data Availability statement on your behalf, as necessary, to reflect the information you provide.***
